# Colloidal Physics Modeling Reveals How Per-Ribosome Productivity Increases with Growth Rate in Escherichia coli

**DOI:** 10.1128/mbio.02865-22

**Published:** 2022-12-20

**Authors:** Akshay J. Maheshwari, Alp M. Sunol, Emma Gonzalez, Drew Endy, Roseanna N. Zia

**Affiliations:** a Department of Bioengineering, Stanford University, Stanford, California, USA; b Department of Chemical Engineering, Stanford University, Stanford, California, USA; Johns Hopkins Bloomberg School of Public Health

**Keywords:** systems biology, colloidal physics, physics of life, molecular crowding, protein synthesis, translation elongation, cytoplasm structure, Brownian motion, computational modeling, translation

## Abstract

Faster-growing cells must synthesize proteins more quickly. Increased ribosome abundance only partly accounts for increases in total protein synthesis rates. The productivity of individual ribosomes must increase too, almost doubling by an unknown mechanism. Prior models point to diffusive transport as a limiting factor but raise a paradox: faster-growing cells are more crowded, yet crowding slows diffusion. We suspected that physical crowding, transport, and stoichiometry, considered together, might reveal a more nuanced explanation. To investigate, we built a first-principles physics-based model of Escherichia coli cytoplasm in which Brownian motion and diffusion arise directly from physical interactions between individual molecules of finite size, density, and physiological abundance. Using our microscopically detailed model, we predicted that physical transport of individual ternary complexes accounts for ~80% of translation elongation latency. We also found that volumetric crowding increases during faster growth even as cytoplasmic mass density remains relatively constant. Despite slowed diffusion, we predicted that improved proximity between ternary complexes and ribosomes wins out, illustrating a simple physics-based mechanism for how individual elongating ribosomes become more productive. We speculate that crowding imposes a physical limit on growth rate and undergirds cellular behavior more broadly. Unfitted colloidal-scale modeling offers systems biology a complementary “physics engine” for exploring how cellular-scale behaviors arise from physical transport and reactions among individual molecules.

## INTRODUCTION

**FIG 1:**
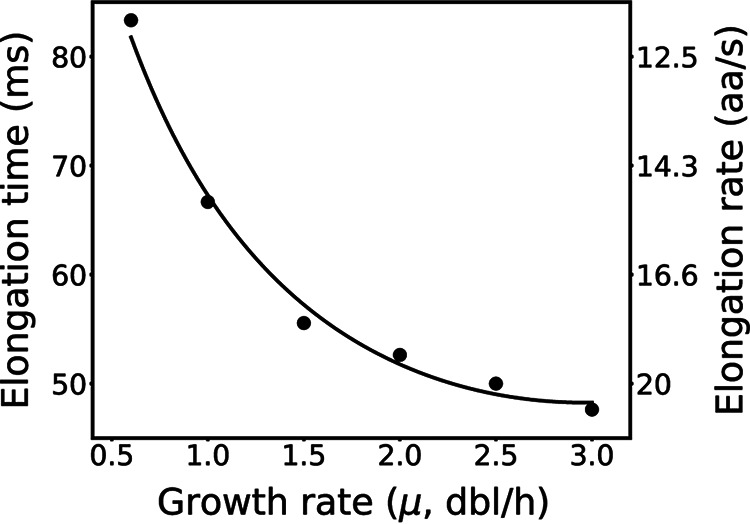
Individual ribosomes make proteins more quickly as growth quickens. Total latency per peptide bond (left *y* axis) and elongation rate (right *y* axis) versus growth rate (*x* axis). Experimental data are from references 1–7. Solid line is a second-order polynomial fit of experimental elongation rates.

Protein synthesis is essential for cell maintenance and reproduction. For example, Escherichia coli cells synthesize the majority of their dry mass as protein every cell doubling. Accordingly, cells that grow more quickly must produce proteins more quickly. In quantitative detail, as E. coli growth speeds up 5-fold, protein synthesis across the entire cell increases 15-fold (1). Meanwhile, for the same growth rate increase, the quantity of ribosomes increases only 9-fold, suggesting that the absolute productivity of individual ribosomes must also somehow increase, almost doubling as growth quickens (Fig. 1 in text, Fig. S1 and Table S1 in the supplemental material at https://doi.org/10.5281/zenodo.7200121) (1–7). While it is easy to understand why having more translation machinery increases total protein synthesis capacity, it is not obvious how faster-growing cells achieve the translation elongation rates needed to sustain growth.

Bremer and Dennis hypothesized that individual ribosome activity speeds up at faster growth rates owing to increased tRNA charging and also due to shifts in codon distribution among mRNA (6). However, subsequent work has shown that overall tRNA charging remains relatively constant across growth rate, indicating that other mechanisms are likely at play (8). Another possibility is that the intrinsic chemical kinetics of peptide bond formation by the ribosome accelerate with increasing growth rates. For example, in exploring how to adapt chemical kinetic rates obtained from *in vitro* experiments for use with *in vivo* models, Rudorf and coworkers fitted parameter values to data and showed that faster chemical kinetic rate constants could account for increased rates of peptide bond formation (9). However, the specific molecular mechanisms that might account for such parameter changes are unknown. As a third possibility, Klumpp and coworkers hypothesized that physical processes could play a limiting role in determining the elongation rate of individual ribosomes (2). More specifically, by accounting for Brownian diffusion of ternary complexes via a growth rate-independent diffusion constant within a Michaelis-Menten kinetics-based model of translation elongation, Klumpp and coworkers inferred that physical changes in cytoplasm could lead to changes in growth rate. Taken together, such studies suggest that both chemistry and physics likely play a role in the speedup of translation elongation.

However, understanding any potential speedup mechanism is challenging exactly because the chemistry and physics of translation elongation are complex and coupled. For example, the biochemical processes required are combinatorial: matching must take place between 42 unique ternary complexes and 64 possible triplet codons. Accordingly, any particular elongating ribosome may encounter numerous mismatching ternary complexes prior to a successful matching reaction. As a second example, the length and time scales of underlying processes span 3 and 9 orders of magnitude, respectively; specifically, ternary complexes and ribosomes interact with surrounding biomolecules and each other over nanometers and nanoseconds but execute processes over micrometers and seconds. As a third example, while higher concentrations of ternary complexes might be expected to increase the frequency of encounters with ribosomes, the resulting increase in crowding might slow the physical search process. Such complexities are compounded by the fact that everything is happening in parallel among hundreds of thousands of self-mixing molecules in a growth rate-dependent and crowded cytoplasm.

We addressed these challenges by modeling both the physics and chemistry of translation elongation in a combined framework. To do so, we adapted an open-source simulation tool (10) to more accurately represent transport and interactions among molecules constituting self-mixing systems. In our framework, we explicitly represent the transport dynamics of individual biomolecules as they physically interact and chemically react, with nanometer and nanosecond resolution, to simulate processes spanning minutes in time. A key aspect of our approach is the robust modeling of Brownian motion and colloidal-scale particle interactions such that these molecules undergo the inertialess physical encounters appropriate to the colloidal regime (11–13). In particular, motion and diffusion rates arise directly in our simulations based on mass, size, and crowding such that resulting emergent behaviors (such as transport and search time) are not merely *ex post facto* fits to expected results. When combined with a well-known multistep kinetic model for the reactions leading to peptide bond formation (14), our framework enables analysis of the combined physical and chemical dynamics underlying translation elongation.

We employed our framework to explore how protein synthesis rates in E. coli should be expected to change with growth rate, directly representing the growth rate-dependent cytoplasm via first-principles modeling of physical and chemical dynamics without parameter fitting. Starting from well-established measurements of macromolecular composition and physical properties of E. coli cytoplasm at various growth rates, we demonstrated how well-known changes in the composition of cytoplasm are entirely sufficient to account for the speedup of translation elongation by individual ribosomes. We also identified the detailed contributions of transport and reaction to total elongation latency by monitoring the trajectories of and reactions between ternary complexes and ribosomes in simulation, finding that transport is the dominant component defining elongation latency. We found that physiological cytoplasmic crowding speeds up the transport mechanism and thus elongation rates overall. We confirmed that the expected speedup due to crowding is insensitive to changes in chemical kinetics needed to exactly match observed elongation rates. Finally, we explored how still-greater crowding, beyond naturally observed limits, should lead to a collapse of the colloidal-scale transport speedup mechanism that ultimately limits the performance of self-mixing living systems.

## RESULTS

### Embedding chemical kinetics within physical transport.

**FIG 2:**
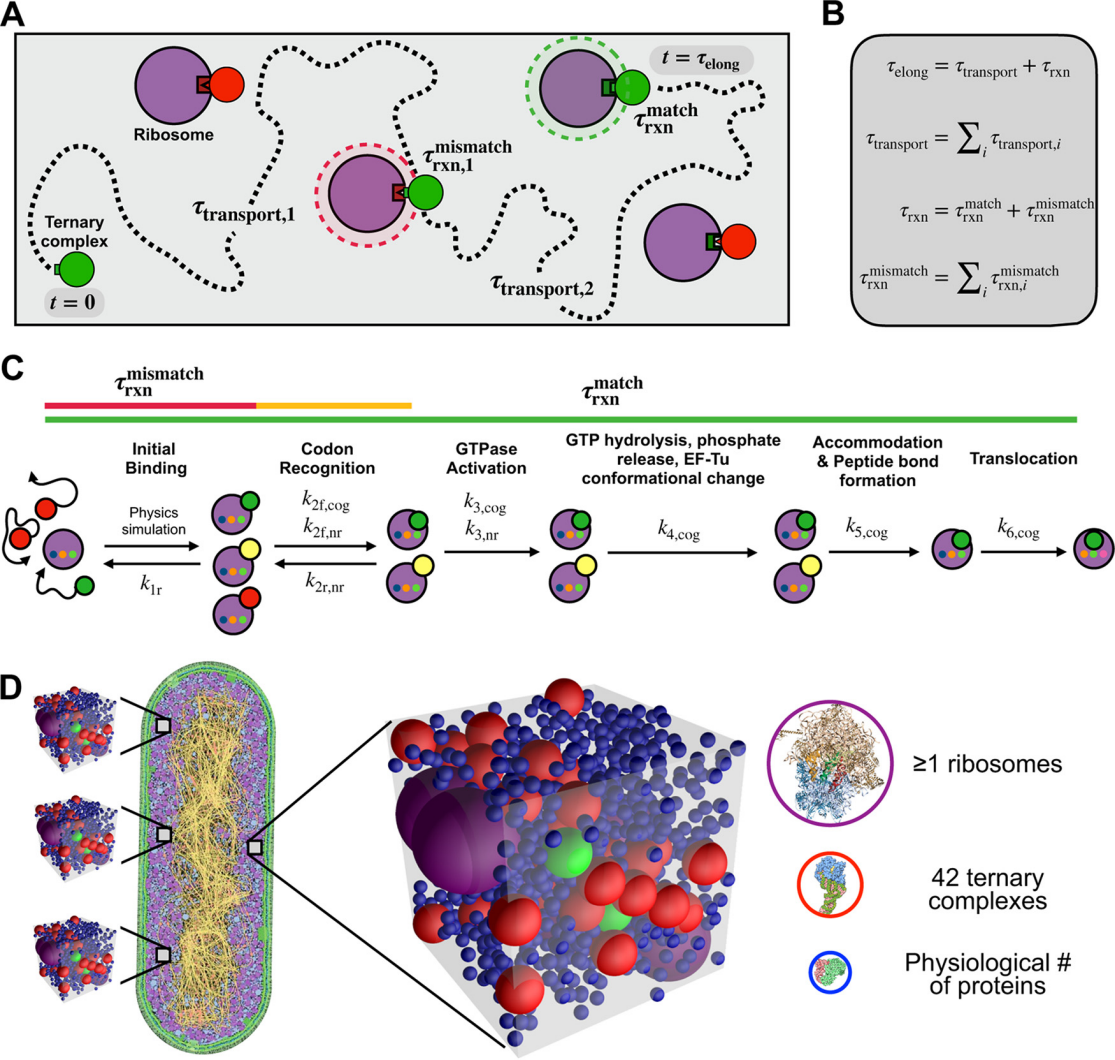
The physical context for translation elongation can be formalized. (A) Schematic of physical and chemical processes that contribute to translation elongation latency. Multiple transport and reaction steps (dashed line) may occur before a ternary complex (green/red) encounters and reacts with an unoccupied, matching ribosome (purple). The time ternary complexes spend unbound while searching for ribosomes is defined as transport latency (τ_transport_), and the time ternary complexes spend bound in either mismatching (red shaded circle) or matching reactions (green shaded circle) is defined as reaction latency (τ_rxn_). The time the entire process takes is defined as elongation latency (τ_elong_). (B) Mathematical definitions of translation elongation latencies. Elongation latency (τ_elong_) is the sum of transport latency (τ_transport_) and reaction latency (τ_rxn_), the latter of which is the sum of both mismatching $\left(\tau_{\mathrm{rxn}}^{\mathrm{mismatch}}\right)$ and matching $\left(\tau_{\mathrm{rxn}}^{\mathrm{match}}\right)$ reaction latencies. (C) Schematic of the kinetic mechanism of translation elongation within ribosomes (purple). Ternary complexes are either cognate (green), near-cognate (yellow), or noncognate (red) to any particular ribosome, which determines kinetic rates. Mismatching reaction latency results from reversible reactions with noncognate and near-cognate ternary complexes (red and yellow lines), while matching reaction latency results from cognate ternary complexes proceeding through the full kinetic process (green line). (D) Translation elongation is evaluated by constructing ensembles of statistically representative “translation voxels” that, in their minimal form, contain exactly 42 ternary complexes (cognate, green; noncognate, red), at least one ribosome (purple), and numerous average-sized proteins representing all other surrounding proteins (blue). The depiction of E. coli is adapted with permission from reference 44; molecular abundances are adapted from the literature (see Results and Materials and Methods).

We constructed a spatially resolved chemical and physical framework to model the combined roles of reaction chemistry and transport physics in translation elongation (Fig. 2A and B), tracking the time spent by ternary complexes unbound and in motion (transport latency [τ_transport_]) as well as reacting with mismatching or matching ribosomes (reaction latency [τ_rxn_]) until a matching reaction is successfully completed. Together, transport latency and reaction latency make up elongation latency (τ_elong_).

To estimate reaction latencies, we represented the molecular reactions between ribosomes and ternary complexes following the individual chemical steps of protein synthesis, accounting for differences due to reactions involving cognate, near-cognate, and noncognate ternary complexes (Fig. 2C). We used well-established *in vitro* kinetic measurements to parameterize our model (Table S5 at https://doi.org/10.5281/zenodo.7200121) and developed physiologically accurate distributions of expected reaction latencies via a Markov process (Appendix SA, Figure S2 at https://doi.org/10.5281/zenodo.7200121). We analyzed the resulting reaction latency distributions, finding that matching reactions between cognate ternary complexes and ribosomes take 42 ms on average when successful (68% probability). We also found that cognate ternary complexes can be rejected (32% probability), in which case reactions take 1.4 ms on average. Mismatching reactions involving near-cognate or noncognate ternary complexes take on average 4.6 ms and 1.4 ms, respectively. We did not consider misincorporation events due to their low overall likelihood (<1% probability).

Next, recognizing that translation elongation takes place within a crowded cytoplasmic milieu, we developed a molecular-mechanistic model for how protein synthesis occurs as a physical process. To start, we estimated the smallest volume of cytoplasm sufficient to enable protein synthesis. We assumed that the molecules required for protein synthesis are homogeneously distributed within the nucleoid-excluded cytoplasm. This volume is ultimately determined by the concentration of ternary complexes as the most limiting species. So defined, each “translation voxel” contains exactly one each of 42 unique ternary complexes, one or more ribosomes, and many native proteins (represented as average-sized molecules that represent all other surrounding proteins) (Fig. 2D).

Brownian diffusion allows each molecule to sample the voxel volume and encounter the others. We modeled diffusion explicitly as a random walk of each of the molecules throughout the voxel, where, due to their finite size, they exclude one another entropically or, in the case of a ternary complex and unbound ribosome pair, initiate a reaction (see Materials and Methods). The benefit of explicit modeling (rather than using prior approaches that insert a diffusion coefficient gleaned from experiments) is that we can follow the detailed motion of each translation molecule in its native molecular context where diffusivity increases or decreases automatically and naturally with changes in abundance and crowding (see Appendix SA in the supplemental material at https://doi.org/10.5281/zenodo.7200121).

Due to nonuniform codon usage and nonuniform relative abundance of each type of ternary complex (Table S6 at https://doi.org/10.5281/zenodo.7200121) as well as stochastic variation in the physical distribution of translation molecules in cytoplasm, for any given cell-wide condition, individual translation voxels should be expected to vary in the exact combinations of unique ternary complexes and elongating ribosomes. For example, a translation voxel might contain more than one of a highly abundant tRNA. Accordingly, starting from our basic translation voxel platform we constructed ensembles of thousands of translation voxels to capture the natural distribution of chemical identities and spatial configurations that, together, better represent the natural variation expected within cytoplasm. We used these more accurate voxel ensembles to examine the physical and chemical mechanistic relationship between growth rate and elongation rate by simulation (see below).

### Stoichiometric crowding accompanies faster protein synthesis.

**FIG 3:**
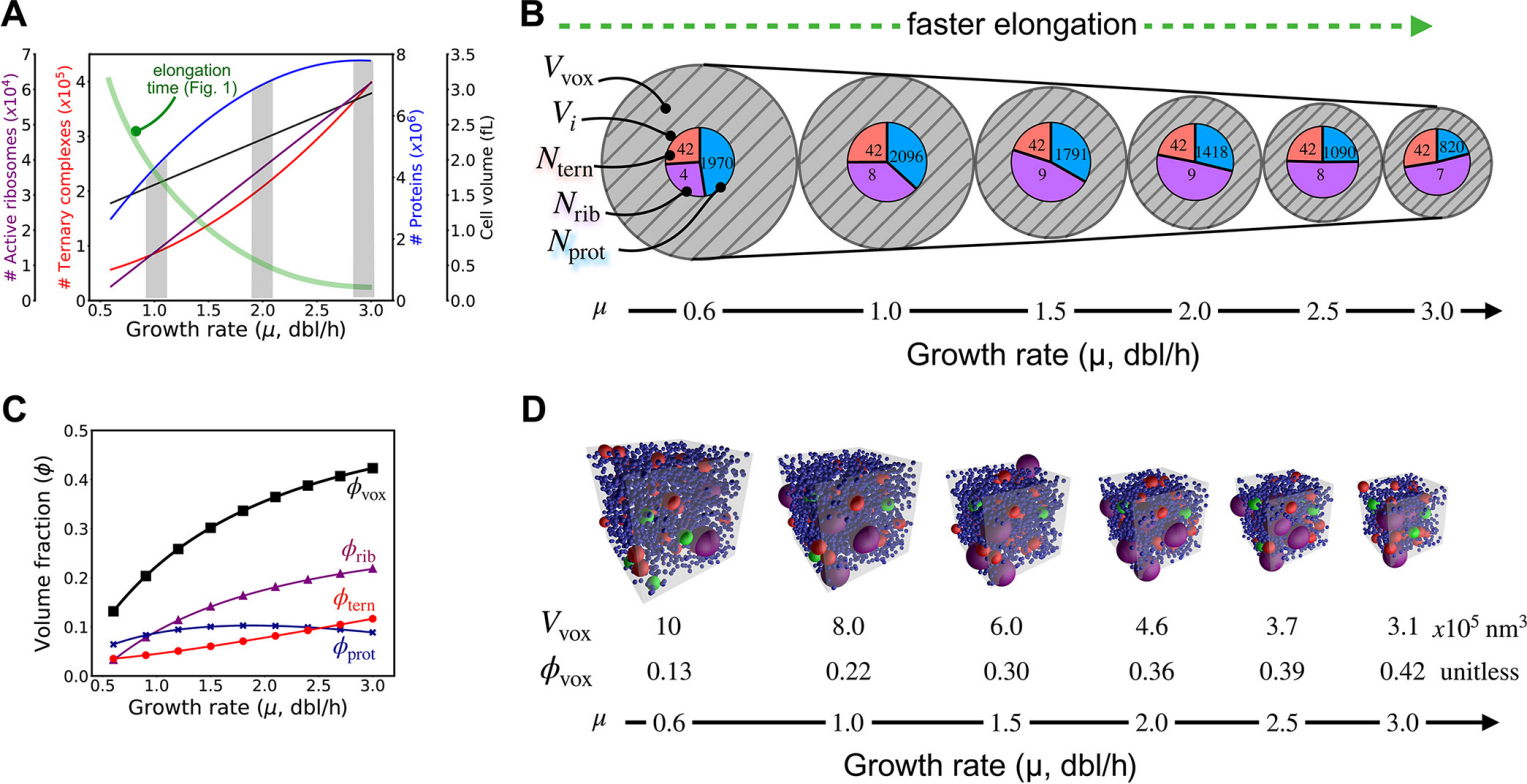
The relative abundances, concentrations, and volume fractions of translation molecules change as growth quickens. (A) Experimental observations of active ribosomes, ternary complexes, and protein abundances in E. coli, as well as E. coli cell volume, reveal varying levels of increase with increasing growth rate (gray bars highlight values at particular growth rates). (B) An abstracted representation of translation voxels as a function of growth rate reveals that differential changes in molecular abundances are accompanied by an overall increase in crowding (i.e., stoichiometric crowding). The volume of translation voxels (*V*_vox_; hatched gray circles) decreases while the total volume of constituent biomolecules (concentric pie charts) remains relatively constant. The total number of each particular type of biomolecule species (*N*_tern_, *N*_rib_, and *N*_prot_), shown within corresponding colors of the pie chart (red, ternary complexes; purple, ribosomes; blue, proteins) in a given translation voxel as well as the total volume each biomolecule species occupies (*V_i_*; the area of corresponding colors within the pie chart) change at different growth rates. (C) The volume fractions (ϕ*_i_* = *V_i_*/*V*_vox_) of ribosomes (ϕ_rib_), ternary complexes (ϕ_tern_), and proteins (ϕ_prot_) change differently with increasing growth rate, leading to an overall increase in the total occupied volume fraction of translation voxels (ϕ_vox_). (D) Representative snapshots of translation voxel simulations at increasing growth rates, along with their respective volumes (*V*_vox_) and volume fractions (ϕ_vox_).

We gathered and analyzed well-established experimental data for cell mass, cell volume, and the sizes and abundances of ternary complexes, ribosomes, and proteins in cells across growth rates (1, 15–19) (see Materials and Methods; also, see Fig. S1 and Tables S1 to S4 at https://doi.org/10.5281/zenodo.7200121). We deduced that, as growth rate (μ) increases (from 0.6 to 3.0 doublings [dbl]/h) and translation elongation speeds up (from 12 to 21 amino acids [aa]/s), ternary complexes and ribosomes monotonically increase in number by nearly an order of magnitude, while proteins monotonically increase by 3-fold (Fig. 3A). We described the coupled abundances and volume fraction of each constituent biomolecule as the “colloidal stoichiometry” of the translation voxel; mathematically, the abundances and volume fraction of each constituent biomolecule *i* are described as *N_i_* and ϕ*_i_*, respectively; ϕ*_i_* is calculated as *V_i_*/*V*_vox_, where *V_i_* is the total volume occupied by a particular biomolecule species and *V*_vox_ is the total volume of the voxel. The colloidal stoichiometry of translation voxels, which captures both chemical and physical features of cytoplasm, changes with growth rate. Thus, we hypothesized that growth rate-dependent changes in colloidal stoichiometry might contribute to the speedup of translation elongation.

More specifically, our modeling revealed changes in the colloidal stoichiometry of translation voxels as growth quickens (from 0.6 to 3.0 dbl/h): voxels shrink 3-fold (i.e., *V*_vox_ decreases from 10E5 nm^3^ to 3E5 nm^3^) and become 3-fold more crowded (ϕ_vox_ increases from 0.13 to 0.42). However, the growth in volume fraction is not uniform across species: as voxels shrink, the number of ribosomes doubles while the number of proteins halves (Fig. 3B and D). That is, increased total crowding is dominated by ribosomes: the volume fraction of ribosomes increases by 7-fold, more than double that of ternary complexes (ϕ_rib_ = 0.03 to 0.22; ϕ_tern_ = 0.04 to 0.12). Proteins dominate total volume fraction at low growth rates but then plateau (ϕ_prot_ = 0.06 to 0.10 for μ values of 0.6 to 2.0 dbl/h; ϕ_prot_ = 0.10 to 0.09 for μ values of 2.0 to 3.0 dbl/h), thus contributing minimally to the overall increase in crowding (Fig. 3C).

We refer to this growth rate-dependent change in colloidal stoichiometry as “stoichiometric crowding,” which we expected would impact both the interactions and motion of translation molecules at different growth rates. For example, in the growth rate trends noted above, as growth quickens, ternary complexes and ribosomes should encounter each other more frequently than they do proteins. As a second example, the distribution of molecule sizes matters: for a fixed total volume fraction, the diffusion of individual particles is faster in a suspension of large than small particles, and as the most dominant particle size shifts from smaller to larger, diffusion of all particles speeds up (21–23); since ribosomes increase in their relative volume fraction with growth rate, voxels should mix more quickly for any given total volume fraction as growth quickens. We remark that the stoichiometric crowding demonstrated here may arise even in the setting of constant overall mass density: in particular, while there have been conflicting reports of overall mass density changing or remaining constant over different ranges of growth rate in *E. coli* (1, 20), molecular abundances and volume fractions change regardless and can impact molecular behavior (see Fig. S18 and Note S5 at https://doi.org/10.5281/zenodo.7200121).

### Physical transport of ternary complexes accounts for most of elongation latency.

We next sought to better understand and quantify any such impacts of crowding and composition on transport rates, reaction rates, and elongation rates overall. To do so, we established a baseline quantification of the relative importance of physical transport to chemical reactions in setting elongation latency. We constructed translation voxels, from very simple to biologically faithful forms, and analyzed expected transport, reaction, and elongation latencies by simulation. From this, we inferred the mechanisms by which colloidal stoichiometry regulates overall elongation latency as a function of growth rate. We used a low growth rate (0.6 dbl/h) as a benchmark, where bulk elongation takes about 87 ms on average (Fig. 1).

**FIG 4:**
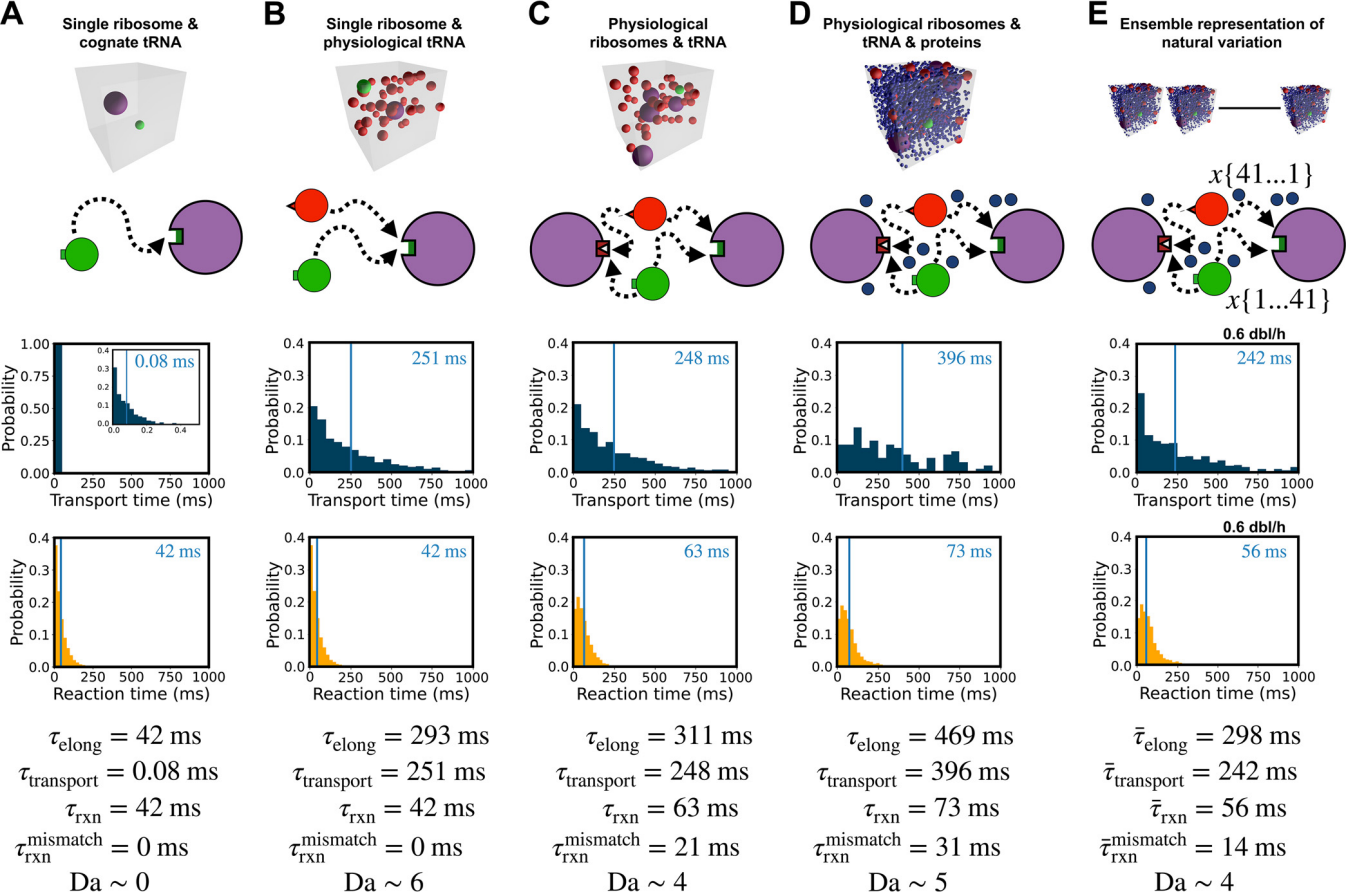
Most of the latency in translation elongation arises from physical transport of ternary complexes. (A to E) Simulation snapshots, model schematics, and simulation results (top to bottom) for increasingly realistic (left to right) translation voxels at a growth rate of 0.6 dbl/h. In each plot, the average latency is marked by a blue vertical line and displayed on the top right in milliseconds. (A) A highly simplified translation voxel containing only a single ribosome and cognate ternary complex. (B) A translation voxel containing a single ribosome and 42 ternary complexes. (C) A translation voxel with 42 ternary complexes and four ribosomes. (D) A translation voxel with 42 ternary complexes, four ribosomes, and 1,970 proteins. (E) An ensemble of translation voxels that capture the expected natural variation in cognate, near-cognate, and noncognate ternary complexes due to nonuniform ternary complex and codon abundances coupled with spatial stochasticity. The standard errors in the estimate of the mean relative to the mean for transport latency and elongation latency are 3% for panels A to C, 9% for panel D, and 6% for panel E, while those for reaction latency are all below 1%.

More specifically, we first studied transport and reaction dynamics in detail using an idealized case in which only a single cognate ternary complex interacts with one matching ribosome (Fig. 4A). Here, there is no competition with mismatching ternary complexes and no other molecules blocking the way, and the sought-after ribosome is unbound; rather a lone ternary complex searches pure cytosolic fluid for a waiting matching ribosome, an idealized scenario often depicted in “textbook” representations of translation elongation (24). As expected, we found that transport latency—the time a ternary complex spends not bound to a ribosome, diffusively searching for a match—is nearly instantaneous ($\tau_{\text{transport}}$ = 0.08 ms) and that nearly all of the elongation process is taken up by reaction latency—the time a ternary complex spends bound to ribosomes ($\tau_{\text{rxn}}$ = 42 ms) (Fig. 4A). While such a result seems to support the conclusion that chemistry alone determines translation elongation rate (Fig. 2C), the notion of a two-molecule translation voxel operating at 3% volume fraction, far below physiological conditions, is unrealistic (Fig. 3C).

We next added a physiologically correct number of ternary complexes to the voxel (i.e., one cognate and 41 noncognate) such that each ternary complex competes to reach and bind to the ribosome (Fig. 4B). We found that the total reaction time remains the same ($\tau_{\text{rxn}}$ = 42 ms). However, the transport latency of the cognate ternary complex increases markedly ($\tau_{\text{transport}}$ = 251 ms) and is greater than reaction latency, supporting arguments that translation elongation requires substantial reactant transport time. The increased transport time is also coupled to reactions: a cognate ternary complex must “wait” to bind with the ribosome while that ribosome is already bound to noncognate ternary complexes; thus, there is an interplay between reactions and transport.

We then added a physiologically correct number of ribosomes to the translation voxel, holding the ternary complex population fixed at one cognate and 41 noncognates (Fig. 4C). Only one ribosome was available for a matching reaction with the cognate ternary complex; the other ribosomes were mismatches for all ternary complexes. Having just one matching ribosome means that we need track only the elongation events of a single ribosome, while simultaneously tracking mismatching events at other ribosomes; this approximation provides a lower-bound estimate for bulk elongation rates while allowing a more accurate accounting of transport and reaction effects (see below). We found by simulation that transport latency remains similar to voxels containing a single ribosome ($\tau_{\text{transport}}$ = 248 ms) because the single matching ribosome remains bound for almost the same amount of time, indicating that the cognate ternary complex still needs to wait nearly as long (i.e., the indirect impact of mismatching reactions). A slight decrease in transport latency can be attributed to some noncognate ternary complexes being bound by mismatching ribosomes, meaning that fewer noncognate ternary complexes are available to occupy the matching ribosome. However, the reaction latency of the cognate ternary complex increases ($\tau_{\text{rxn}}$ = 63 ms) due to the direct impact of mismatching reactions: cognate ternary complexes spend more time in futile interactions with the more abundant mismatching ribosomes.

Next, we added a physiologically correct abundance of proteins to the translation voxel, resulting in further increases in both transport and reaction latencies ($\tau_{\text{transport}}$ = 396 ms;$\tau_{\text{rxn}}$ = 73 ms) (Fig. 4D). These predicted increases arose because proteins increase the number of mismatching reactions by trapping noncognate ternary complexes near ribosomes, which in turn both promotes repeated mismatch reactions and reduces cognate ribosome availability (Fig. S6 at https://doi.org/10.5281/zenodo.7200121).

Finally, we represented the expected statistical variation in cytoplasm by constructing thousands of different voxels that, together, capture the physiological distribution of relative abundances of translation molecules as reported in the literature (see Materials and Methods). Specifically, in E. coli, there are 42 unique ternary complexes, each with its own abundance, and 64 codons, each with its own usage rate, and these are present in many permissible combinatoric configurations in translation voxels throughout the cytoplasm (Fig. 2D). We randomly sampled all permissible configurations using reported E. coli codon usage and whole-cell tRNA abundances (see Materials and Methods; also, see Table S6 and Fig. S4 at https://doi.org/10.5281/zenodo.7200121). Recognizing that bulk elongation measurements correspond to the time needed to complete as many successful reactions as there are ribosomes in a voxel, we computed the transport, reaction, and elongation latencies of each translation voxel from the time taken for a single matching reaction within the voxel. A weighted average of the per-ribosome latency for all permissible translation voxels corresponds to the typical time for just one matching reaction to occur in a voxel and thus provides a lower-bound estimate of bulk experimental elongation time as obtained from cellular measurements (see Materials and Methods). While our prior simulations (Fig. 4A to D) included only noncognate and cognate ternary complexes, our calculations of the ensemble latencies (Fig. 4E) also included the more detailed classification of some ternary complexes as near-cognate, which affects system dynamics further because near-cognates are well known to have a longer rejection time than noncognates (Table S5; Fig. S2 at https://doi.org/10.5281/zenodo.7200121). We monitored transport, reaction, and elongation latency during simulation in each of these thousands of voxels and computed a weighted-average value for transport, reaction, and elongation latency. We found that the weighted-average transport, reaction, and elongation latencies decrease in the ensemble representation (${\bar{\tau }}_{\text{transport}}$ = 242 ms; ${\bar{\tau }}_{\text{rxn}}$ = 56 ms; ${\bar{\tau }}_{\text{elong}}$ = 298) (Fig. 4E), compared to the single-voxel simulation (Fig. 4D). This across-the-board decrease in latencies emerges naturally from the majority of voxels in which there is more than one cognate ternary complex, partly a result of the biological phenomenon of more frequently used codons being associated with more abundant cognate tRNAs (Fig. S8 at https://doi.org/10.5281/zenodo.7200121).

Quantitatively, the Damköhler number (Da)—the ratio of the latency of transport to reaction ($\tau_{\text{transport}}$/$\tau_{\text{rxn}}$)—highlights the dependency of translation elongation latency on physical transport relative to chemical reaction. In our simplest model, Da is ~0, suggesting that reaction latency dominates elongation latency. However, our increasingly accurate models estimate Da values of 6, 4, 5, and 4, respectively (Fig. 4B to E). We thus concluded that processes that modulate transport latency play a dominant mechanistic role in regulating the overall speed of translation elongation.

### Stoichiometric crowding speeds up translation elongation.

We returned to the puzzle of what mechanism(s) might cause the productivity of individual ribosomes to increase with increasing growth rates. We first evaluated the impact of stoichiometric crowding on transport latency by considering both molecule proximity (i.e., how close molecules are to one another) and molecular mobility (i.e., how fast molecules move). We also evaluated the impact of stoichiometric crowding on reaction latency by considering both local availability (i.e., the extent to which ternary complexes are free from repeated mismatching reactions) and global availability (i.e., the extent to which ternary complexes are free from mismatching reactions generally). Taking these data together, we determined if and how each of these coupled physicochemical mechanisms might regulate elongation latency as a function of growth rate.

**FIG 5:**
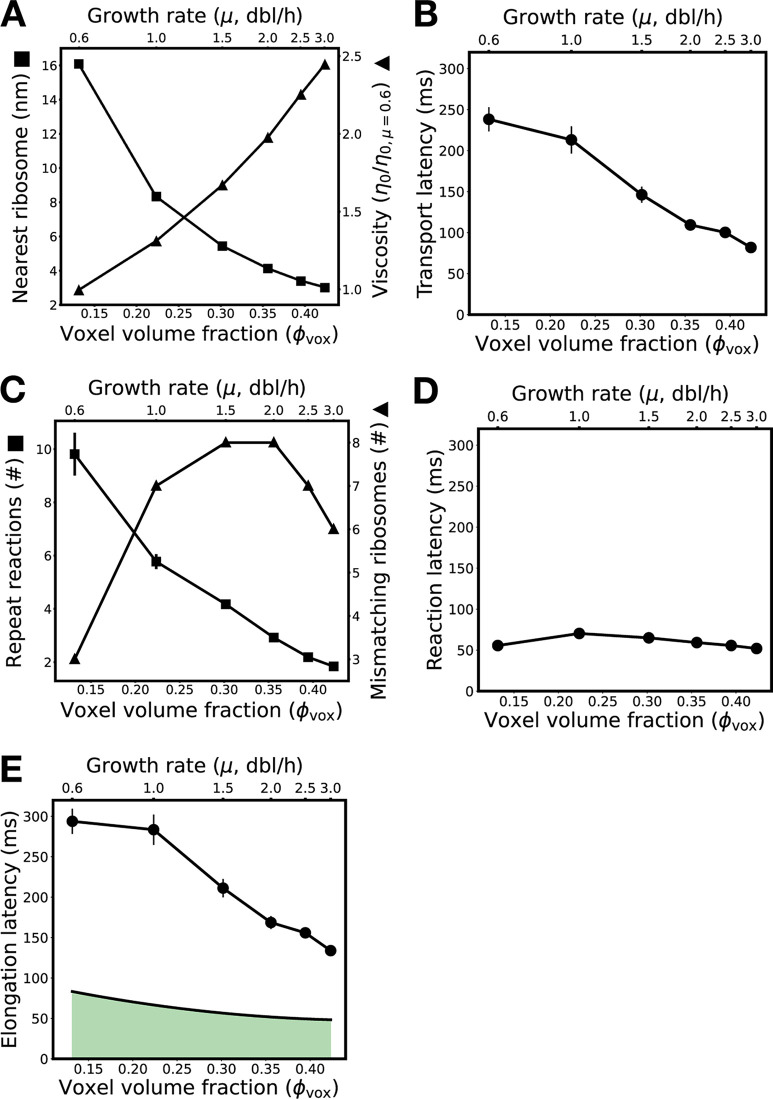
Stoichiometric crowding reduces both intermolecular distances and transport latency, resulting in increasingly productive ribosomes as growth rate increases. (A) As crowding and growth rate increase (*x* axes), ternary complexes become closer to their nearest ribosome (left *y* axis) and translation voxel viscosity increases (right *y* axis). Distance is reported as a surface-to-surface estimate. Viscosity is reported normalized to viscosity at a growth rate of 0.6 dbl/h. (B) Simulation results showing that transport latency (*y* axis) decreases with increased crowding and growth rate (*x* axes). (C) As crowding and growth rate increase (*x* axes), the average number of repeat reactions between ternary complexes and ribosomes decreases (left *y* axis), while the absolute number of mismatching ribosomes in a translation voxel first increases then decreases (right *y* axis). (D) Simulation results showing that reaction latency (*y* axis) first increases then decreases with increased crowding and growth rate (*x* axes). (E) Simulation results showing that the predicted absolute elongation latency decreases with increased crowding and growth rate (*x* axes). Experimentally measured per-ribosome elongation latency (solid line upon green area; replotting of Fig. 1) also speeds up with growth rate but is faster than predicted across all growth rates. The standard errors in the estimate of the mean for all model results (A to E) are shown (error bars).

We hypothesized that crowding should tend to reduce transport latency because ternary complexes need to search smaller volumes to find a matching ribosome. To explore this idea, we computed the average surface-to-surface distance between ternary complexes and their closest ribosomes (i.e., the shortest distance a ternary complex needs to travel to find a ribosome) across hundreds of translation voxels at multiple growth rates (see Materials and Methods). We found that stochiometric crowding brings ternary complexes and ribosomes 5-fold closer on average (16 nm to 3 nm), which supports our hypothesis (Fig. 5A, left axis). However, stoichiometric crowding could also increase transport latency as ternary complexes become hindered in their motion and thus take longer to search. To explore this second idea, we examined the influence of stoichiometric crowding on molecule mobility by estimating the viscosity of cytoplasm as well as the hindered diffusivity of ternary complexes, ribosomes, and proteins via simulation of hundreds of translation voxels at multiple growth rates (Fig. 5A, right axis; also, see Fig. S3 at https://doi.org/10.5281/zenodo.7200121 and Materials and Methods). We found that stochiometric crowding increases viscosity monotonically (1.0 to 2.4, normalized to viscosity at a μ of 0.6 dbl/h) while reducing diffusivity monotonically for all biomolecules (e.g., the diffusivity of ternary complexes, *D*_tern_, slows from 35 μm^2^/s to 16 μm^2^/s), which would support the opposite conclusion: that crowding should hinder transport. Recognizing this competition between an increased proximity reducing transport latency and an increased viscosity increasing transport latency, we more systematically considered the contribution of each mechanism to transport as stoichiometric crowding increased due to increased cell growth rate.

For example, we simulated ensembles of translation voxels representing the full statistical distribution of ternary complexes and codon abundances from low to high growth rates (see Materials and Methods). We found that transport latency monotonically decreases with stoichiometric crowding ($\tau_{\text{transport}}$ = 242 ms to 83 ms) (Fig. 5B). We deduced that, mechanistically, crowding drives faster transport because reducing the search distance between ternary complexes and ribosomes is more important than increased viscosity. However, while this net decrease in transport latency will decrease elongation latency overall, it could be that coupled changes in reaction latency either reverse or reinforce this trend.

Thus, we examined two mechanisms that could modulate reaction latency. First, we recalled that proteins can induce repeat reactions by trapping ternary complexes and ribosomes together (see “Physical transport of ternary complexes accounts for most of elongation latency”). These repeat reactions should reduce the local availability of ternary complexes, making it more difficult for ternary complexes to find matching ribosomes, driving up reaction latency. To examine whether increased stoichiometric crowding amplifies this effect, we tracked the number of times ternary complexes consecutively rereact with the same ribosome following a mismatching reaction across hundreds of translation voxels at various growth rates. We were surprised to find that repeated reactions decrease 5-fold (from ~10 to ~2 repeat reactions per ribosome on average) as growth rate quickens and total crowding increases (Fig. 5C, left axis). We resolved this apparent paradox by recognizing that the increased crowding arises primarily due to tighter packing of ribosomes, while the volume fraction of proteins hardly changes. We deduced that this provides more local ribosome alternatives (i.e., higher local availability) for ternary complexes but with no increase in trapping by proteins (Fig. S6 at https://doi.org/10.5281/zenodo.7200121). However, having more ribosomes, regardless of how well packed they are, provides more opportunities to preoccupy ternary complexes in mismatch reactions, reducing ternary complex global availability, which should drive up reaction latency. We found that the number of mismatching ribosomes in a voxel first increases and then decreases with growth rate, which should contribute an initial increase and then decrease in reaction latency as growth rate increases (Fig. 5C, right axis). Taken together, the observations indicate that the total impact on reaction latency depends on the relative strengths of each of these effects.

We next computed reaction latencies across our ensembles of translation voxels, capturing how physiological variation influences the competition between local and global availability. We found that total reaction latency increases (${\bar{\tau }}_{\text{rxn}}$ = 56 ms to 70 ms) at low but increasing growth rates and then decreases monotonically thereafter (${\bar{\tau }}_{\text{rxn}}$ = 70 ms to 56 ms) (Fig. 5D). Low global availability of ternary complexes dominates at low growth rates, slowing reaction latency as growth rate increases. However, at higher growth rates, the increases in both global and local availability combine to drive down reaction latency. Overall, the growth rate trend in reaction latency (Fig. 5D) follows ternary complex global availability (Fig. 5C, right axis). Practically, even with a 25% increase that subsequently reverses, reaction latency changes only 7% over low to high growth rates, suggesting that transport plays the more substantial role in speeding elongation.

Indeed, the quantitative speedup of reaction latency with growth rate (Fig. 5D) is minor compared to the corresponding speedup of transport latency (Fig. 5B), indicating that transport mechanisms should be expected to dominate over reaction mechanisms in regulating the growth rate-dependent productivity of individual ribosomes. Our ensemble simulations show that the dominance of transport manifests in the total elongation latency as a monotonic speedup of elongation with growth rate (${\bar{\tau }}_{\text{elong}}$ = 298 ms to 135 ms), recovering the experimental trend of faster elongation at higher growth rates ($\tau_{\mathrm{elong}}^{\mathrm{bulk}}$ = 83 ms to 48 ms) (Fig. 5E).

Finally, although our model correctly predicts and recovers the qualitative behavior and overall trend (i.e., an increase in ribosome productivity with increasing growth rate), we noted that our unfitted bottom-up modeling and simulations result in absolute predictions of translation elongation latencies that are ~3-fold too slow compared to experimental observations (Fig. 5E). Thus, we conducted sensitivity analyses in which chemical kinetic rates were fitted to match observed overall translation elongation latency (see Note S1 at https://doi.org/10.5281/zenodo.7200121). We found that the speedup in translation elongation is insensitive to changes in the chemical kinetics of translation elongation (Fig. S13 at https://doi.org/10.5281/zenodo.7200121).

## DISCUSSION

The observed increase in individual ribosome activity as growth quickens has not been explained mechanistically (Fig. 1). While many have focused attention on how translation initiation contributes to protein synthesis latency, we were intrigued by how the individually fast steps of translation elongation add up during translation elongation and should dominate overall process latency (Note S2 at https://doi.org/10.5281/zenodo.7200121). In the context of translation elongation, while prior studies of protein synthesis rates focused on chemical kinetic measurements or kinetics-based modeling, there have been persistent signals that Brownian diffusion of translation molecules plays a role in setting elongation rates.

With this in mind, we explored the idea that either chemistry or physics, or both, contributes to the speedup of translation elongation utilizing dynamic simulations. We proposed that reactions between ternary complexes and ribosomes are nontrivially coupled to their physical transport and that understanding this coupling is essential to explaining increased ribosome productivity at higher growth rates. To systematically interrogate the role of coupled physicochemical processes in translation elongation, we adapted an open-source simulation tool to accurately represent transport of and interactions between translation molecules in cytoplasm. A key aspect of our approach is the robust modeling of Brownian motion and colloidal-scale particle interactions such that these molecules undergo the inertialess physical encounters appropriate to the colloidal regime. We defined translation voxels as naturally emergent from the constituent biomolecules required for translation and captured the natural distribution of chemical identities and spatial configurations of translation molecules in cytoplasm by constructing ensembles of thousands of voxels. We monitored in simulations the reactions and transport of molecules in these voxel ensembles to study the physical and chemical mechanistic relationships between growth rate and elongation rate (Fig. 2).

We found that transport latency—the time ternary complexes spend searching for cognate ribosomes—is an essential component of elongation latency. Furthermore, we predicted that transport latency dominates over reaction latency—the time ternary complexes spend reacting with ribosomes (Fig. 4). Indeed, physical transport of individual ternary complexes accounts for ~80% of elongation latency. By examining the elongation process as growth rate increases, we identified two competing mechanisms that underlie transport latency: proximity between ternary complexes and ribosomes, which sets search distance, and cytoplasmic crowding, which sets diffusive speed. Additionally, we observed that translation molecules become 3-fold more crowded with increasing growth rate, suggesting that, beyond any absolute increase in the abundance of translation machinery, the machinery itself becomes packed closer together. The abundance and packing are physical as well as chemical (i.e., colloidal stoichiometry), and their changing due to changes in growth rate is a phenomenon we call stoichiometric crowding (Fig. 3). Overall, we found that increased packing at higher growth rates improves proximity, which, along with changes in stoichiometry, increases the frequency of cognate reactions; this in turn increases individual ribosome productivity (Fig. 5), revealing a mechanistic explanation for why individual ribosomes can produce proteins more quickly in faster-growing cells.

Our colloidal-scale, mechanistic conclusions complement existing phenomenological modeling work that describes how protein synthesis and growth can be predicted by resource allocation kinetics and optimization (2, 25, 26). We also help resolve a paradox arising from the observations by Klumpp et al., which suggested that slower diffusion of ternary complexes leads to slower growth but then cannot explain faster growth occurring under more crowded conditions (2). Here, we explain how slower diffusion accompanies faster growth, which is to be expected given increased crowding in faster-growing E. coli (Fig. 5; also, see Note S4 at https://doi.org/10.5281/zenodo.7200121). Translation speeds up as diffusion goes down: closer proximity (i.e., molecules closer together at higher crowding) outpaces reduced diffusivity, resulting in faster translation elongation and thus faster growth overall. Crowding favors ribosomes and translation molecules at higher growth rates: the more crowded cytoplasm has a higher relative abundance of translation molecules than other proteins (Fig. 3). The resulting improved proximity and changed stoichiometry speed up translation.

We stress-tested our model and confirmed that the speedup of elongation requires physical transport and that our prediction of speedup is robust to changes in the values of the input chemical parameters. We also found that increasing 3-fold the values for all nine *in vitro* literature values for chemical kinetics parameters closes the quantitative gap between predicted and observed elongation rates (Fig. S13 at https://doi.org/10.5281/zenodo.7200121). This suggests the rather straightforward chemistry-only explanation for the gap: *in vitro* measurements being “off” by ~300%, uniformly across all nine parameters. But, interestingly, we also found that a 30-fold increase in only the ternary complex unbinding rate (*k*_1r_)—the only reaction that takes place exclusively outside the ribosome—could also close the quantitative gap (Fig. S9 at https://doi.org/10.5281/zenodo.7200121), suggesting that there may be a mechanism involved *in vivo* that quickens ternary complex exchange or one that obviates fast rejection (i.e., a mechanism for favoring matching reactions near to the ribosome).

Overall, our model reveals new opportunities for discovery. For example, better representation of electrostatic and hydrodynamic interactions or detailed molecular shape and orientation for site specificity may be useful. More specifically, attractive interactions between the ribosomal L7/L12 domain and ternary complexes (27) or between cognate ternary complexes and mRNA (28) could have the effect of preloading or presorting ternary complexes. As a second example, hydrodynamic models of small and large particles confined in a cavity show that both types of particles tend to concentrate near the cavity surface with minor impact on the mobility of small particles (23), indicating that ternary complexes and ribosomes may concentrate by the cell membrane (not currently represented in our model) and effectively improve in proximity to each other.

More generally, our work supports exploration of the role of coupled, colloidal-scale physicochemical interactions in cytoplasm. For example, we predicted that ternary complexes and ribosomes would be up to 5-fold closer together in faster-growing cells (Fig. 5A), a major shift in the colloidal-scale structure of cytoplasm that can be expected to modulate molecular interactions broadly across the cytoplasm. Such colloidal-scale structure is being increasingly measured experimentally (e.g., ribosome spatial positioning via cryo-electron tomography of entire cells) and merits increased attention for its role in cytoplasm behavior (29, 30). As a second example, the phenomenon of repeat reactions that we found to be critical to the speedup of protein synthesis has also been identified as critical to efficient activation of mitogen-activated protein (MAP) kinases (31), suggesting a wider role for repeat reactions in cell functions. One can also infer the possibility that stoichiometric crowding with changing growth rate may impact cell signaling in general. As a third example, using our model we predicted that the viscosity of the nucleoid-excluded cytoplasm increases up to 2.5-fold with growth rate, which indicates a decrease in the mobility of all constituent molecules. Such a broad growth rate-dependent shift in colloidal-scale dynamics may suggest currently unappreciated forms of physical regulation in cells and motivates a renewed analysis of diffusive processes in cells with consideration of volume fraction and growth rate.

Broadly, a more complete understanding and representation of the colloidal-scale dynamics that underlie cellular processes can offer a practical first-principles foundation for systems biology and whole-cell modeling (32–34). Advances in both computational modeling and experimental technique are needed to improve the accuracy of our predictions and promote broader exploration of how coupled colloidal-scale physics and chemistry in cytoplasm might regulate cellular behaviors. For example, dynamic simulation of the motion of solvent-suspended particles requires discretization of the time domain, where equations of motion are integrated forward in time. The selection of time step size impacts not only computational expense but also the fidelity of particle encounters where, for example, too-large time steps can produce pathological displacements in response to steeply attractive or repulsive forces (e.g., the hard-sphere repulsion that represents entropic exclusion is singular at particle contact), a phenomenon that becomes more severe as crowding increases. Here, we performed a careful study to prioritize physical accuracy first and then optimize efficiency by, for example, developing a kinetic scaling method that leveraged the natural disparity between diffusive and reactive time steps (Materials and Methods; Fig. S3, S5, S10, and S14 at https://doi.org/10.5281/zenodo.7200121). Even so, further capturing the complexity of cytoplasm (e.g., protein polydispersity, general protein-protein interactions, polysome dynamics, or cell cycle dependency) will ultimately require modeling other microscopic forces at play in cytoplasm, including electrostatic or hydrodynamic interactions or membrane confinement, all of which lead to many-body interactions that increase computational expense. Modeling such forces, in addition to other molecular details like shape, softness, flexibility, and site specificity, is becoming possible with other algorithms, such as Stokesian dynamics for large or confined systems (23, 35–38), but will require substantial integration and iteration with experiments as well as improvements in computational efficiency to achieve accurate simulations over the timescales of cellular behavior (39). Capturing detailed molecular dynamics, such as those involved with ternary complex-ribosome binding and reactions, will also necessitate multiscale modeling and experimentation from atomic to cellular scales.

**FIG 6:**
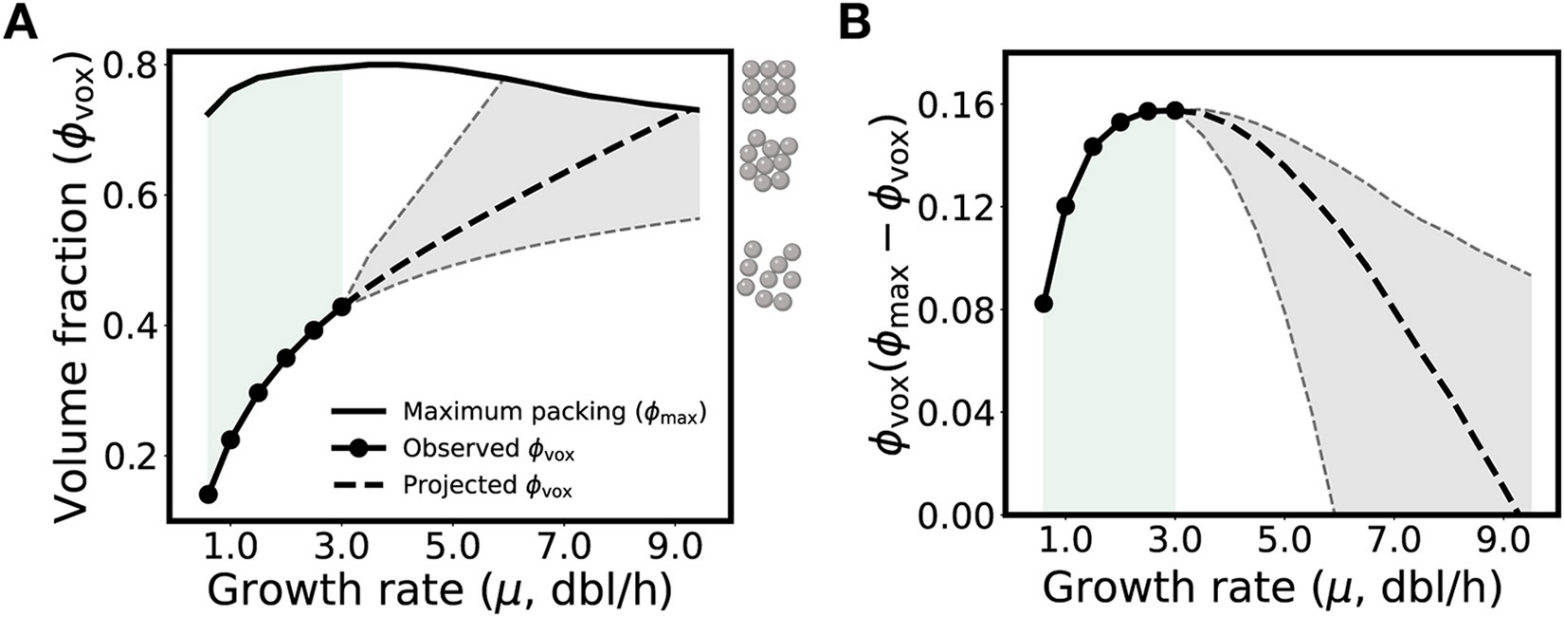
Stoichiometric crowding has diminishing returns that may impose a physical limit on growth rate. (A) Volume fraction of translation voxels at observed growth rates and projected growth rates compared to theoretical maximum random close packing (maximum packing changes with size polydispersity [43] and size polydispersity changes with growth rate). Bounds for volume fraction at projected growth rates are shown (gray dashed line and shading). Maximum packing increases across observed growth rates (green shading). The volume fraction for the most-crowded observed voxel (ϕ_vox_ = 0.42; μ = 3.0 dbl/h) is shown (right axis, bottom schematic). For reference, the volume fractions at which long-term molecular motion in monodisperse suspensions is hindered or halted due to random close packing (ϕ = 0.64) or crystallization (ϕ = 0.74), respectively, are also shown (right axis, middle and top schematic). (B) The product of voxel volume fraction (ϕ_vox_) and remaining available volume fraction (ϕ_max_ − ϕ_vox_) increases across observed growth rates (green shading) before decreasing across higher-than-observed growth rates.

To conclude, we note that protein synthesis is inextricably tied to growth rate and fitness; cells cannot grow more quickly than they can reproduce their proteome, including the proteins that remake the proteome. Since stoichiometric crowding facilitates faster protein synthesis, could further crowding enable still-faster growth or, conversely, limit how fast cells can grow? To speculate, as a simple extension of our modeling, we projected what cytoplasm of faster-than-observed growing E. coli would look like (Fig. S11 at https://doi.org/10.5281/zenodo.7200121; also, see Materials and Methods). We found that E. coli would eventually reach maximum packing (i.e., with little to no space for molecular mixing) as growth rate continues to increase (Fig. 6A). That such hypothetical growth rates are not observed suggests, among other possibilities, that as growth rate increases beyond the maximum observed, the beneficial effects of increased crowding (e.g., increased proximity) become outpaced by the deleterious effects of less available free volume (e.g., further increased viscosity) (Fig. 6B). Our theoretical observation expands upon a past proposal that growing cells optimize total protein volume fraction for both reaction and transport (40) and hints that stoichiometric crowding may be linked to fitness and evolutionary fine tuning (Note S3 at https://doi.org/10.5281/zenodo.7200121). If so, then we would expect that genes encoding currently unknown functions may serve to establish a physical, as well as chemical, basis for fitness (e.g., proteome polydispersity) undergirding cellular behavior broadly.

## MATERIALS AND METHODS

### Construction of a representative translation voxel.

We developed computational representations of translation voxels by analyzing the abundances and sizes of molecules contained in E. coli cytoplasm in relation to overall cell volume and mass. Where needed, we inferred abundances across growth rates by fitting polynomials to reported measurements (see below).

**(i) Calculation of biomolecular abundances in cells.** We computed the average abundances of ribosomes (*N*_rib_) and ternary complexes (*N*_tern_) in single cells across physiological growth rates using existing data from literature (Table S1 at https://doi.org/10.5281/zenodo.7200121). To compute the abundance of proteins surrounding ribosomes and ternary complexes, we first calculated the total dry mass of proteins in cytoplasm, *M*_cytoplasm,prot_ (equation 1). Protein mass encompasses the mass of all biomolecules in cytoplasm other than ribosomes, ternary complexes, mRNA, and DNA:

(1) $$\begin{array}{c} M_{\mathrm{cytoplasm},\mathrm{prot}}=M_{\mathrm{cytoplasm}}-M_{\mathrm{tern}}\times N_{\mathrm{tern}}-M_{\mathrm{rib}}\times N_{\mathrm{rib}}-M_{\mathrm{cytoplasm},\;\mathrm{mRNA}}-M_{\mathrm{cytoplasm},\mathrm{DNA}}\; \end{array}$$

The masses of ternary complexes (*M*_tern_) and ribosomes (*M*_rib_) were specified using known molecular structures and the total mass of mRNA, DNA, and cytoplasm (*M*_cytoplasm,mRNA_, *M*_cytoplasm,DNA_, and *M*_cytoplasm_, respectively) were taken from literature (Tables S1 and S4). We then calculated the average effective spherical radius of proteins (${\bar{R}}_{\mathrm{prot}}$), as well as the mass of resulting average-sized proteins (${\bar{M}}_{\mathrm{prot}}$), using single-cell E. coli mass spectrometry data and average protein density (ρ_prot_) (ProteinComputation.ipynb):

(2) $$\begin{array}{c} V_{\mathrm{prot},i}\;=\frac{M_{\mathrm{prot},i}}{\rho_{\mathrm{prot}}} \end{array}$$

(3) $$\begin{array}{c} {\bar{R}}_{\mathrm{prot}}\;=\underset{i}{\sum }\frac{N_{\mathrm{prot}-\mathrm{poly},i}}{N_{\mathrm{prot}-\mathrm{poly}}}\;\sqrt[{3}]{\frac{3V_{\mathrm{prot},i}}{4\pi }} \end{array}$$

(4) $$\begin{array}{c} {\bar{M}}_{\mathrm{prot}}\;=\frac{4}{3}\pi ({\bar{R}}_{\mathrm{prot}}^{3})\left(\rho_{\mathrm{prot}}\right) \end{array}$$

We obtained the mass and abundances of each protein in the E. coli cytoplasm (*M*_prot,_*_i_* and *N*_prot-poly,_*_i_*, respectively), as well as the total number of proteins (*N*_prot-poly_), from the mass spectrometry measurements of Schmidt and colleagues (18). We also computed the volume of each protein in the E. coli cytoplasm (*V*_prot,_*_i_*) using these measurements. We then determined the abundance of proteins in a cell as the ratio of total mass occupied by proteins in cytoplasm (equation 1) and the mass of average-sized proteins (Fig. S1B at https://doi.org/10.5281/zenodo.7200121),

(5) $$\begin{array}{c} N_{\mathrm{prot}}=\frac{M_{\mathrm{cytoplasm},\mathrm{prot}}}{{\bar{M}}_{\mathrm{prot}}} \end{array}$$

We note that the size polydispersity of proteins is weak, meaning that they do not deviate much from the average size (Fig. S16 at https://doi.org/10.5281/zenodo.7200121). Although we show that the presence of proteins deeply influences translation dynamics (Fig. 4; Fig. S3, S6 at https://doi.org/10.5281/zenodo.7200121), the fact that proteins are typically much smaller than ribosomes and ternary complexes means that protein size variation is a vanishingly small influence in comparison. As a result, we expect negligible change to ternary complex dynamics and overall translation rates, justifying our representation of protein crowding with an average size.

**(ii) Calculation of translation voxel size and biomolecular abundances in translation voxels.** On a relative basis, amino acid-specific ternary complexes are the least concentrated molecules involved in translation elongation, and thus, their concentration determines the minimum volume of cytoplasm capable of supporting protein synthesis. Therefore, we determined the size of translation voxels from our estimates of ternary complex abundances (*N*_tern_) and E. coli volume (*V*_cell_) across growth rates (Fig. S1; Tables S1 and S2 at https://doi.org/10.5281/zenodo.7200121). More specifically, we defined a translation voxel to be the volume of cytoplasm that contains 42 ternary complexes (*N*_tern,vox_ = 42), assuming a spatially homogeneous distribution of ternary complexes within cytoplasm:

(6) $$\begin{array}{c} V_{\mathrm{vox}}=\frac{N_{\mathrm{tern},\mathrm{vox}}\times V_{\mathrm{cell}}}{N_{\mathrm{tern}}}\; \end{array}$$

We then calculated the average number of proteins (*N*_prot,vox_) and ribosomes (*N*_rib,vox_) within the translation voxel volume (*V*_vox_) assuming a homogeneous distribution of both species, but with ribosomes excluded by the nucleoid (Fig. S1C at https://doi.org/10.5281/zenodo.7200121):

(7) $$N_{\mathrm{prot},\;\mathrm{vox}}=\frac{N_{\mathrm{prot}}\times V_{\mathrm{vox}}}{V_{\mathrm{cell}}}\;$$

(8) $$\begin{array}{c} N_{\mathrm{rib},\;\mathrm{vox}}=\frac{N_{\mathrm{rib}}\times V_{\mathrm{vox}}}{V_{\mathrm{cell}}(1-\phi_{\mathrm{nucleoid}})} \end{array}$$

We estimated the volume fraction of the nucleoid (ϕ_nucleoid_) as the volume of the nucleoid relative to cell volume based on published values (Table S3 at https://doi.org/10.5281/zenodo.7200121).

**(iii) Polynomial regression fitting.** We computed polynomial fits for ribosome abundances, ternary complex abundances, cell mass, cell volume, and nucleoid volume fraction using a bootstrapping method that minimized the mean absolute error (Fig. S1A at https://doi.org/10.5281/zenodo.7200121; TranslationVoxelParameterization.ipynb); we used mean absolute error instead of mean squared error to penalize all variation equally.

### Calculation of translation voxel volume fractions and polydispersity.

We calculated the volume fraction of ribosomes, ternary complexes, and proteins (Fig. 3C) using ribosome, ternary complex, and protein abundances in translation voxels; ribosome, ternary complex, and average-sized protein single-molecule volumes; and translation voxel volume:

(9) $$\begin{array}{c} \phi_{\mathrm{rib}}=\frac{N_{\mathrm{rib},\mathrm{vox}}\times V_{\mathrm{rib}}}{V_{\mathrm{vox}}}\; \end{array}$$

(10) $$\begin{array}{c} \phi_{\mathrm{tern}}=\frac{N_{\mathrm{tern},\mathrm{vox}}\times V_{\mathrm{tern}}}{V_{\mathrm{vox}}}\; \end{array}$$

(11) $$\begin{array}{c} \phi_{\mathrm{prot}}=\frac{N_{\mathrm{prot},\mathrm{vox}}\times {\bar{V}}_{\mathrm{prot}}}{V_{\mathrm{vox}}}\; \end{array}$$

The abundances of ribosomes, ternary complexes, and proteins (*N*_rib,vox_, *N*_tern,vox_, and *N*_prot,vox_) in translation voxels as well as the translation voxel volume (*V*_vox_) are as described in equations 6, 7, and 8. The volume of a single average-sized protein (${\bar{V}}_{\mathrm{prot}}$) is defined as its mass divided by average protein density (equation 4). We computed the volume of single ribosomes (*V*_rib_) and ternary complexes (*V*_tern_) based on their longest length (i.e., estimating the molecules as spheres with diameters equal to the longest length of the molecules), which we measured from their detailed atomic resolution structures (PDB 4V4Q and PDB 1B23, respectively) (Table S4 at https://doi.org/10.5281/zenodo.7200121).

We calculated the size polydispersity, s, of translation voxels as in reference 41:

(12) $$\begin{array}{c} \;s=\sqrt{\frac{\sum^{\;}N_{i,\mathrm{vox}}\times \sum^{\;}N_{i,\mathrm{vox}}R_{i}^{2}}{{\left(\sum^{\;}N_{i,\mathrm{vox}}R_{i}\right)}^{2}}-1}\; \end{array}$$

Here, *N_i_*_,vox_ and *R_i_* correspond to the translation voxel abundances and effective spherical radius, respectively, of each biomolecule type, denoted by the subscript *i* (Fig. S1, Table S4 at https://doi.org/10.5281/zenodo.7200121).

### Simulation of translation voxels.

We simulated transport and reaction of biomolecules within translation voxels using Brownian dynamics and single-molecule reaction kinetics, respectively. We implemented our simulations using “Colloidal Smoldyn,” our adaptation of the open-source simulation software Smoldyn (10). Colloidal Smoldyn accurately represents single-molecule resolution colloidal transport dynamics and reaction dynamics as described in detail in Appendix SA at https://doi.org/10.5281/zenodo.7200121. The time step we used in our simulations is Δ*t* = 62 ps.

### Simulation of translation voxels with varying composition.

To measure the relative contributions of transport and reactions to protein synthesis rate, we simulated translation voxels with increasingly accurate composition (μ = 0.6 dbl/h) (see “Physical transport of ternary complexes accounts for most of elongation latency” in Results; ColloidalStoichiometryEffects.ipynb). Specifically, we simulated five progressively accurate scenarios: (i) a single ribosome and cognate ternary complex matching pair; (ii) a matching pair surrounded by a physiological number of ternary complexes; (iii) a matching pair surrounded by physiological numbers of ternary complexes and ribosomes; (iv) a matching pair surrounded by physiological numbers of ternary complexes, ribosomes, and proteins; and (v) a statistically representative ensemble of translation voxels each with a physiological number of ternary complexes, ribosomes, and proteins. We performed 900 simulation replicates each for the first three cases and 100 simulation replicates for case 4; we detail case 5 in the next section. Replicates were assigned random initial conditions, chosen using a Mersenne Twister random number generator with seeds set at multiples of five (i.e., 0, 5, 10, …). Since reaction kinetics inside the ribosome (following codon recognition) are well known and unidirectional (Fig. 2C; Fig. S1, Table S4 at https://doi.org/10.5281/zenodo.7200121), we did not explicitly model intraribosomal kinetics within voxel simulations. Instead, we modeled reaction kinetics following codon recognition separately by producing and sampling from a distribution of 30,000 post-codon recognition reaction times with the kinetic rates summarized in Table S5 at https://doi.org/10.5281/zenodo.7200121.

### Construction of statistically representative translation voxel ensembles.

To construct statistically representative ensembles of translation voxels (see Results), we incorporated reported relative abundances of different types of ternary complexes and frequencies of codons among mRNA in E. coli at different growth rates (Table S6 at https://doi.org/10.5281/zenodo.7200121). We computationally constructed 100,000 translation voxels for cells in each of six different growth conditions (0.6, 1.0, 1.5, 2.0, 2.5, and 3.0 dbl/h), sufficient to represent the statistical distribution of translation voxels across each condition. Individual translation voxels comprise different types of ternary complexes and codon-specific elongating ribosomes, randomly chosen using a Mersenne Twister random number generator with a seed of zero.

For each translation voxel, we randomly picked a single ribosome to track. We then classified ternary complexes in each translation voxel as either noncognate or cognate to the chosen ribosome, leading to a growth rate-dependent distribution in the abundance of cognate ternary complexes (between 0 and 42) across translation voxels (Fig. S4 at https://doi.org/10.5281/zenodo.7200121, CognatetRNADistributionCalculation.ipynb). For each of our six modeled growth conditions, we used the distribution of cognate ternary complexes calculated at the closest growth rate (measured at 0.4, 0.7, 1.07, 1.6, and 2.5 dbl/h). The speed with which the single chosen ribosome in a translation voxel successfully finds and reacts with a cognate ternary complex provides a good lower bound for the bulk translation elongation rate; the bulk elongation rate corresponds to the speed with which as many peptide bonds are formed as there are ribosomes, and the speed with which a single ribosome finds and successfully reacts with a cognate ternary complex will typically be higher than the speed of as many successful reactions as ribosomes in the voxel.

### Simulation of statistically representative translation voxels ensembles.

To compute the transport, reaction, and elongation latencies of statistically representative ensembles of translation voxels (see Results), we simulated translation voxels across the six different growth conditions (0.6, 1.0, 1.5, 2.0, 2.5, and 3.0 dbl/h). Statistically representative ensembles of translation voxels correspond to the full set of possible translation voxels, meaning that translation voxels can contain 0 to 42 cognate ternary complexes for a single chosen ribosome (distributed as in Fig. S4 at https://doi.org/10.5281/zenodo.7200121). For translation voxels containing 1 to 42 cognate ternary complexes belonging to cells growing at each of the six growth rates, we simulated 100 replicates starting from different random initial conditions (42 × 6 × 100 = 25,200 total simulations). We set conditions for each replicate using the Mersenne Twister random number generator with seeds set as multiples of five (i.e., 0, 5, 10, …, 495). Simulations were terminated when the ribosome being tracked successfully reacted with a cognate ternary complex.

### Post-simulation analysis of statistically representative translation voxel ensembles.

For each translation voxel simulation (*i*), we computed the elongation latency (τ_elong,_*_i_*), transport latency (τ_transport,_*_i_*), and reaction latency (τ_rxn,_*_i_*) of the cognate ternary complex that successfully reacted with the ribosome being tracked (StatisticallyRepresentativeTranslationVoxelAnalysis.ipynb). We incorporated the impact of near-cognate ternary complexes by scaling the time taken by a statistically accurate portion of noncognate reactions at the tracked ribosome. Specifically, we leveraged our calculations that translation voxels have eight near-cognate ternary complexes and 32 noncognate ternary complexes, on average (Fig. S4 at https://doi.org/10.5281/zenodo.7200121) and that near-cognates have an average latency of 4.6 ms while noncognates have an average latency of 1.4 ms (Fig. S2 at https://doi.org/10.5281/zenodo.7200121), to randomly scale noncognate reaction times 3.3-fold with 20% probability. We note that this representation of near-cognates does not capture the impact of near-cognate ternary complexes on other ribosomes in the voxel; near-cognates could slightly reduce overall latencies by occupying mismatching ribosomes for longer than noncognate ternary complexes, allowing cognate ternary complexes to find their match more quickly.

We subsequently computed an overall transport latency (${\bar{\tau }}_{\text{transport}}$), reaction latency (${\bar{\tau }}_{\text{rxn}}$), and elongation latency (${\bar{\tau }}_{\text{elong}}$) for each growth rate. We did so by calculating weighted averages of each of transport latency, reaction latency, and elongation latency acquired from translation voxel simulations for each particular growth rate (μ), averaging over replicates (*j*):

(13) $$\begin{array}{c} {\bar{\tau }}_{\text{elong}}\left(\mu \right)=\frac{1}{100}\overset{42}{\underset{i}{\sum }}\overset{100}{\underset{j}{\sum }}p_{i}\tau_{\mathrm{elong},i} \end{array}$$

(14) $$\begin{array}{c} {\bar{\tau }}_{\text{transport}}\left(\mu \right)=\frac{1}{100}\overset{42}{\underset{i}{\sum }}\overset{100}{\underset{j}{\sum }}p_{i}\tau_{\mathrm{transport},i} \end{array}$$

(15) $$\begin{array}{c} {\bar{\tau }}_{\text{rxn}}\left(\mu \right)=\frac{1}{100}\overset{42}{\underset{i}{\sum }}\overset{100}{\underset{j}{\sum }}p_{i}\tau_{\mathrm{rxn},i} \end{array}$$

The probability of each translation voxel configuration (*p_i_*) is conditional on both the number of cognate ternary complexes in the particular translation voxel and growth rate (Fig. S4 at https://doi.org/10.5281/zenodo.7200121). We did not consider the latency of translation voxels that contained zero cognate ternary complexes (~22% of translation voxel instances), since such voxels would have infinite latency and are an artifact of constraining translation voxels to 42 total ternary complexes. In particular, if larger voxels with more than 42 ternary complexes are considered, the resulting proportion of cognate ternary complexes is similar but with fewer instances of zero cognates (e.g., we found that voxels with 42, 84, or 168 ternary complexes have the same number of average cognates when normalized by number of total ternary complexes but have 22%, 10%, and 3% instances with zero cognates, respectively). Not considering the zero cognate ternary complex voxels thus provides a lower bound estimate of elongation, transport, and reaction latency.

### Event-based stochastic simulations of statistically representative translation voxel ensembles.

To measure the effect of removing transport physics from our simulations (Note S1, Fig. S13, S6, S7 at https://doi.org/10.5281/zenodo.7200121), we developed an event-based stochastic simulation algorithm of statistically representative translation voxel ensembles (EventBasedStochasticSimulation.ipynb). As in our other simulations, the ensemble of voxels captures the relative abundances of ternary complexes and frequencies of codons among mRNA, but unlike in our other simulations, physical space is not represented.

In our stochastic simulation algorithm, all ribosomes in translation voxels are initialized as bound to randomly chosen ternary complexes. Each reacting ternary complex-ribosome pair is then assigned a time until either disassociation or successful amino acid incorporation, drawn from the distribution of noncognate, near-cognate, and cognate reaction latencies we computed (Fig. S2 at https://doi.org/10.5281/zenodo.7200121). The simulation proceeds in an event-based fashion, iteratively transitioning to the next event that occurs in the translation voxel (i.e., the time step of simulation is not fixed). Following a disassociation event, the disassociated ternary complex joins the available (unbound) ternary complex population, and the newly available ribosome instantly binds to a randomly chosen ternary complex. The simulation ends when a cognate ternary complex successfully reacts with a matching ribosome.

We computed the elongation latency at particular growth rates by simulating the statistical distribution of possible translation voxels (i.e., with the full permissible range of cognate ternary complexes, distributed as in Fig. S4 at https://doi.org/10.5281/zenodo.7200121) and then averaging their resulting elongation latencies. For each growth rate and permissible number of cognate ternary complexes, we simulated 5,000 replicate translation voxels with different random initial conditions. Conditions were set for each replicate using the Mersenne Twister random number generator with seeds set as multiples of five (i.e., 0, 5, 10, …, 24,995).

### Computational tools and costs.

All fixed time-step simulations of translation voxels were performed using Colloidal Smoldyn (based on Smoldyn v2.61) deployed on Amazon Web Services. Simulations required ~300,000 CPU-hours in total. Our longest simulations, for translation voxels at a growth rate of 0.6 dbl/h, took up to ~3 weeks for some replicates, while our shortest simulations took seconds. The cost of all our simulations was approximately US $10,000. Output file sizes for most simulation runs were small (<1 MB). All measurements and validation with the LAMMPS Molecular Dynamics Simulator were performed using the National Science Foundation's XSEDE high-performance computational resources and Stampede2 cluster at the Texas Advanced Computing Center (TACC). Modeling, analysis, and event-based simulations were performed using Python 3.7.

### Acceleration of translation voxel simulations to reduce run time and cost.

Simulations of translation voxels were originally forecast to cost US $6 million with the longest simulations taking ~36 years, making them intractable. To achieve feasible costs and run times, we implemented a procedure for accelerating our fixed-time-step simulations ~600-fold, reducing costs and run times as detailed above. In our acceleration procedure, kinetic rates of unbinding and codon recognition (i.e., the possible exits to the initially bound state) are increased 600-fold during simulations (*k*_1_ = 717 s^−1^ to 430,200 s^−1^ and *k*_2f_ = 1,474 s^−1^ to 884,400 s^−1^). Simulations are run until completion following a successful match between a cognate ternary complex and matching ribosome. Subsequently, during post-simulation analysis, the time spent by ternary complexes in the initially bound state is rescaled to be 600-fold longer, and rescaled times are used to compute reaction, transport, and elongation latencies. Reaction latency is calculated as the time the cognate ternary complex spends bound in reactions, elongation latency is calculated as the total time the matching ribosome spends unbound or bound in reactions, and transport latency is calculated as the difference between elongation latency and reaction latency.

Our estimates of overall reaction, transport, and elongation latencies are not sensitive to this scaling procedure at the ~600-fold acceleration used (Fig. S5A to C at https://doi.org/10.5281/zenodo.7200121). This insensitivity is a result of unbinding kinetics remaining slow enough that, for a certain range of kinetic scaling, ternary complexes mix within the translation voxel between unbinding events to a sufficiently similar extent (Fig. S5D at https://doi.org/10.5281/zenodo.7200121).

### Calculation of long-time self-diffusivity.

We tracked the motion of individual biomolecules as they wandered far from their original positions, executing a random walk through the cytoplasm. This sampling of many configurations in a voxel is termed the long-time self-diffusion ($D_{\infty }^{s}$) (referred to as diffusivity in Results) and is a monotonically decreasing function of volume fraction at fixed molecule size polydispersity. We computed the long-time self-diffusion of particular biomolecule species (denoted by a subscript *i*) at different growth rates by tracking the absolute position of biomolecules and computing their mean squared displacement over time (see “Stoichiometric crowding speeds up translation elongation” in Results):

(16) $$\begin{array}{c} D_{\infty ,i}^{s}=\frac{1}{6}\lim_{t\to \infty }\frac{d}{\mathrm{dt}}\langle \Delta x_{i}(t)\cdot \Delta x_{i}(t)\rangle \; \end{array}$$

Here, the angle brackets signify an ensemble average over the motion of every biomolecule of a given species in a translation voxel and $\Delta x_{i}$ is the total displacement of a particle from its initial position over time *t*.

### Calculation of viscosity.

We calculated the viscosity of translation voxels at different growth rates (see “Stoichiometric crowding speeds up translation elongation” in Results) by performing shear rheology simulations in LAMMPS. For each growth rate, we initialized suspensions representative of multiple contiguous translation voxels. We imposed a simple shear flow on the suspensions at a constant shear rate in the *x* direction $\left({\dot{\gamma }}_{x}\right)$ and measured the resulting interparticle stress (${\bar{\sigma }}_{\mathrm{xy}}^{p}$). The shear rate imposed was chosen to be small enough to remain in the linear-response regime (i.e., with insignificant deformation), allowing measurement of the intrinsic or so-called zero-shear viscosity (η_0_) normalized here by the solvent viscosity η (ViscosityCalculation.ipynb) (42):

(17) $$\begin{array}{c} \frac{\eta_{0}}{\eta }=1+\frac{5}{2}\phi +\frac{{\bar{\sigma }}_{\mathrm{xy}}^{p}}{\eta {\dot{\gamma }}_{x}} \end{array}$$

Here, the first two terms on the right-hand side of the equation are the Einstein viscosity, and they approximate the hydrodynamic contribution of particles to viscosity at equilibrium. The third term describes the interparticle contribution to viscosity and is equivalent to the Green-Kubo equilibrium interparticle contribution at the small shear rates used here. ${\bar{\sigma }}_{\mathrm{xy}}^{p}$ is computed as the *xy* component of the interparticle stress ($\langle xF^{p}\rangle$), where *x* corresponds to the position vectors of the particles, ***F^p^*** is the (negative of) the gradient of a nearly hard-sphere, spherically symmetric repulsive potential, and the angle brackets signify an ensemble average over all interactions in a translation voxel.

### Calculation of molecular proximity.

We computed the proximity between ternary complexes and ribosomes at different growth rates (see “Stoichiometric crowding speeds up translation elongation” in Results). For each growth rate, we initialized 100 translation voxels with random initial spatial configurations, chosen using a Mersenne Twister random number generator with seeds set at multiples of five (i.e., 0, 5, 10, …, 495). Following a brief equilibration period, we measured the distance from each ternary complex to its closest ribosome. Our reported values of proximity for any particular growth rate are averages of the minimal distance for all ternary complexes across all corresponding translation voxel replicates.

### Calculation of repeat reactions.

We computed the average number of repeat reactions between ternary complexes and ribosomes at different growth rates (see “Stoichiometric crowding speeds up translation elongation” in Results). For each growth rate, we initialized 100 translation voxels with random initial spatial configurations, chosen using a Mersenne Twister random number generator with seeds set at multiples of five (i.e., 0, 5, 10, …, 495). We subsequently tracked the number of times a ternary complex consecutively rereacted with the same ribosome following a mismatching reaction within each translation voxel. Our reported values for repeat reactions for any particular growth rate are averages across all corresponding translation voxel replicates.

### Chemical kinetics sensitivity analysis.

To measure the sensitivity of our predicted elongation latencies to changes in chemical kinetics, we simulated the impact of decreasing or increasing intraribosomal kinetic rates on elongation latency. To do so, we simulated ensembles of translation voxels as described above while varying kinetic rates individually or together and measuring the resulting elongation latency (Fig. S9 at https://doi.org/10.5281/zenodo.7200121). Since the ternary complex unbinding rate (*k*_1r_) impacts the mixing time of voxels (Fig. S5 at https://doi.org/10.5281/zenodo.7200121), we varied the level of kinetic acceleration in our simulations for different values of *k*_1r_, ensuring that our translation voxels were simulated in regimes in which elongation latency is insensitive to changes in kinetic acceleration (our kinetic acceleration scheme is described above).

### Calculation of maximum packing and projected growth rate voxel parameters.

We computed the theoretical maximum packing for translation voxels between observed growth rates, 0.6 dbl/h to 3.0 dbl/h, as well as higher hypothetical growth rates, 3.0 dbl/h to 8.0 dbl/h, using the theoretical calculations of maximum packing for tridisperse systems of Farr and Groot (43). Our translation voxels are composed of molecules having a 1:3:6.5 size ratio, which differs from the particle size ratio used by Farr and Groot (1:3:9) (43), giving an overprediction of our computed maximum packing of less than 10%.

To estimate ribosome abundances, ternary complex abundances, cell mass, cell volume, and nucleoid volume fraction at hypothetical growth rates between 3.0 dbl/h and 8.0 dbl/h, we extrapolated from observed growth rates (Fig. S1 at https://doi.org/10.5281/zenodo.7200121), guided by observed trends below 3.0 dbl/h and allowing uncertainty while rejecting unphysical projections (e.g., negative cell mass and nucleoid volume fraction) (Fig. S11 at https://doi.org/10.5281/zenodo.7200121; Fig. 6). We calculated bounds by perturbing the extrapolated fits while still maintaining all expected trends (e.g., the lower bound of ribosome abundances never decreases with increasing growth rate). We computed volume fractions for translation voxels at hypothetical growth rates as described above, setting upper and lower bounds by considering all permutations of fits for ribosome abundances, ternary complex abundances, cell mass, cell volume, and nucleoid volume fraction (ProjectedGrowthRateCalculations.ipynb).
